# Experiences and coping behaviours of adolescents in Pakistan with alopecia areata: An interpretative phenomenological analysis

**DOI:** 10.3402/qhw.v10.26039

**Published:** 2015-01-29

**Authors:** Rafia Rafique, Nigel Hunt

**Affiliations:** 1 Institute of Applied Psychology, University of the Punjab, Lahore, Punjab, Pakistan; 2 Division of Psychiatry and Applied Psychology, School of Medicine, University of Nottingham, Nottingham, UK

**Keywords:** Adolescents, semi-structured interviews, interpretative phenomenological analysis, coping behaviour, alopecia areata

## Abstract

The study explored experiences of adolescents aged 15–19 with alopecia areata (AA) and investigated their accounts of coping behaviours. Interpretative Phenomenological Analysis was used to provide an in-depth and holistic perspective of their accounts. Semi-structured interviews were undertaken with a volunteer sample of eight respondents diagnosed with AA. Four key themes were identified: loss (self/social), concerns (physical/future), negative (emotions/thoughts), and coping styles (adaptive/maladaptive). Females experienced greater feelings of loss, were more concerned about their looks and their future, and reported more negative thoughts and emotions. Females felt angry and blamed God for their fate; males blamed both their fate and luck. Action-oriented and practical coping styles were adopted by all of them. After the realization that initial coping behaviours were ineffective, self-distraction, acceptance, and humour were used. Psychological relief followed with the practice of religion and planning for treatments to be undertaken in the future. The findings here are similar to research conducted in the West, though with more emphasis on religion. Health care providers and student counsellors need to understand the negative psychosocial consequences for adolescents living with a visible disfigurement and provide appropriate psychological and social support.

Scalp hair has greater psychological and social importance than biological significance. Alopecia areata (AA) refers to unexpected patchy hair loss and can result in a distinctive change in appearance. Alopecia totalis refers to loss of all scalp hair, and alopecia universalis to the loss of all body hair. Here we refer to these three forms as AA. The experience of AA leads to numerous personal, social, and occupational problems (Hunt & McHale, 2005).

Adolescence is a time of internal turmoil and upheaval, and having to face a visible disfigurement at this transitional period can be extremely challenging. Adolescence is a period of major physical changes and emotional turmoil which can lead to reduced self-confidence, shyness, and anxiety resulting in academic, personal, and social pressures (Shulman, Carlton-Ford, Levian, & Hed, 1995). Having to also live with a physically altering condition as AA may bring additional psychosocial concerns in these adolescents.

Adolescents and adults with AA have psychological concerns such as anxiety, depression, low self-esteem, and poor body image (McGarvey, Baum, Pinkerton, & Rogers, 2001). Ruiz-Doblado, Carrizosa, and Hernandez (2003) also report a high prevalence of mood, adjustment, and depressive and anxiety disorders. A systematic review of 19 studies, on a total sample of 1271 participants, established there are negative psychological issues in patients with AA, particularly regarding “self-esteem, body image, and/or self-confidence,” (Tucker, 2009).

There are individual differences in coping styles in adolescents with AA. Some adolescents are likely to adopt adaptive styles, whereas others tend to use maladaptive styles of coping (Cartwright, Endean, & Porter, 2009; Garcia, 2010). Cash, Santos, and Williams (2005) discussed three aspects of body image coping: avoidance, appearance fixing, and positive rational acceptance, suggesting that such strategies develop in conditions that are likely to threaten or affect an individual's self-concept and body image.

Because of the unpredictable nature of AA, emotional coping and social support, as compared to problem-oriented coping strategies, play a significant role in dealing with hair loss (Stowell, Kiecolt-Glaser, & Glaser, 2001). Social situations may be anticipated as being fearful and create avoidance which could in turn generate more fear (Newell, 2000). Children with AA find it challenging to handle bullying and ridicule at school (Hunt & McHale, 2005). Adolescents feel uncomfortable revealing their appearance-related concerns to peers which further produces feelings of isolation.

Constant negative thoughts and language create anxiety, worry, and self-blame, all negative types of coping (Garcia, 2010). Coping style depends on sex and age; age and ethnicity have also been shown to mediate coping behaviours in adolescents with AA (Wilson, Pritchard, & Revalee, 2005).

Seeking support from family and friends is effective in dealing with appearance-related concerns of people with a visible disfigurement (Rumsey, Clarke, White, Wyn-Williams, & Garlick, 2004). Thompson, Kent, and Smith (2002) propose that living with an appearance-changing illness is not linear and incremental. Factors such as personality traits, severity, and duration of the condition play a vital role with respect to the adaptation and coping process. Bereavement for one's previous appearance is a part of adaptation and coping. A number of psychological adjustments are desirable before one can start reconciling with the altered appearance (Papadopolous & Bor, 1999).

There are sex differences in the use of coping strategies by adolescents (Frydenberg & Lewis, 1996). Women face more negative psychosocial consequences with hair loss. Hair loss results in significant deterioration in women's self-concept as compared to men (Freedman, 1994).

The aim of this study is to gain an understanding of adolescents’ personal lived experiences of AA. It explores how adolescents’ accounts indicate ways in which they attempt to make sense of their experiences and how they use coping behaviours to adapt to their condition.

Previous research has not attempted to capture a holistic perspective of the lived psychosocial experience of adolescents with AA. The purpose of using Interpretative Phenomenological Analysis (IPA) was to engage with the meaning that these experiences hold for the participants, by considering researchers’ own conceptions. We used semi-structured interviews with adolescents diagnosed with AA to investigate their psychological and social experiences and accounts of coping behaviours. The study addressed the following research questions.

General question: How do adolescents with AA make sense of their experiences and cope with AA?

Specific question I: How is the experience of adolescents with AA related to the psychosocial context?

Specific question II: What are the behaviours adopted by adolescents to cope with their experience of AA?

## Method

### Research design

#### Interpretative phenomenological analysis

IPA focuses on lived experience of participants by incorporating dual components: phenomenology and interpretation. First it shares the aims of the idiographic phenomenology, which provides detailed analysis of elements of the reflective personal and subjective view of individual experiences. IPA moves one step beyond phenomenology (participants account) and attempts to report on the participant's experience by considering the researchers own view of the world (researcher's interpretations). It recognizes the researcher within the research and analytic process. IPA is based on the principle of double hermeneutic: the researcher is trying to make sense of the participants’ attributed meaning of events, experiences, and states. Interpretations are based on the researcher's own conceptions, beliefs, expectations, and experiences (Smith, Jarman, & Osborne, 1999).

IPA requires reflexivity from the researcher who is expected to explicitly present his or her own perspectives, thus illuminating the analysis (Willig, 2001). Phenomenological research in its true sense requires participants to engage with these reflexive techniques in order to give a more accurate representation of the way in which they perceive and interpret their world (Caelli, 2000).

Smith et al. (1999) describe two approaches to IPA. The first is the basic method, termed the ideographic case study approach. This method is suitable for small samples and enables the researcher to write up a single case or an exploration of themes shared between cases. The other approach is recommended for larger sample sizes and for exploring patterns and relationships within and between conceptual groups.

The ideographic case study approach was used in this study for exploration of themes shared between cases. This approach is suggested for the exploration and development of in-depth descriptions from a single case or shared themes from up to 10 cases (Smith et al., 1999). The ideographic case study approach utilizes interview data, which is audio recorded and transcribed verbatim. When the purpose of the research is to explore the respondents’ perceptions of what is important in relation to the phenomenon in question, set predefined questions are not usually used. It is helpful to note down non-verbal communication. The researcher notes down general impressions of issues such as the tone of the interview and the respondent's ability to retrieve information for discussion. These observations are helpful when interpreting the data (Smith, Flowers, & Larkin, 2009). IPA is inductive, allowing the unanticipated to emerge. Smith believes that being inductive is a central feature of IPA.

IPA was initially adopted within the domain of health psychology (Flowers, Smith, Sheeran, & Beail, 1997; Osborn & Smith, 1998) in order to analyse qualitative data reflecting participants’ experience.

The study was led by IPA to provide an in-depth and sexwise holistic perspective to address the research questions (Smith, 2004; Smith & Osborn, 2003).

#### Procedure

The study was approved by the ethical review boards of the relevant private clinic and hospitals, and the parent university. Participants were recruited with the help of dermatologists working in the research units of the dermatology departments of three hospitals and one private clinic. The informed consent form and participant information sheet made clear that the interviews would be audio recorded. Participants were assured that their participation was voluntary and they were free to leave the study for any reason at any time. Confidentiality and anonymity was assured. Participants were interviewed by the first author either in the hospital or at their home; all interviews were conducted in a manner that ensured privacy. The interviewees felt comfortable with the interviewer as rapport was developed, no reduced expression was observed due to sex difference between the interviewer and the interviewee. The transcriptions were translated into English from the native language (*Urdu*). Data were anonymized at the point of transcription. The mean interview duration was 45 min. In case of ambiguity or lack of clarity during the interview the interviewer asked the interviewee to clarify during the interview. Transcripts were not returned for comments to he interviewees as it was a onetime interview only. Reflective memos comprised personal reactions and subjective reflections were kept for facilitation at the latter stages of analyses.

#### Participants

A volunteer sample of eight adolescents, who were formally diagnosed with AA, had hair loss at the time of interview, and who had a visible disfigurement for between 1 and 3 years (to allow enough time for living with the experience of alopecia) were recruited (see Table I for key characteristics). They were interviewed to explore their “lived experiences” of the condition. Adolescents with other types of alopecia (such as androgenetic or chemotherapy-induced alopecia) were excluded as those forms progress in a manner that is more predictable. Adolescents with AA and a comorbid psychiatric disorder, mainly generalized anxiety disorder, depression, and phobic states were also excluded from participation. None of the participants had any form of physical disability. Out of 10 participants who fulfilled the study inclusion criteria, eight volunteered to participate in the study. These criteria are in line with IPA, which generally uses small homogeneous samples, and participants are recruited through convenience sampling (Smith, 2004). Explicit and thoughtful selection of participants was carried out according to the study's inclusion/exclusion criterion that is in line with the purpose of the study (Smith & Osborn, 2003). IPA researchers generally work with homogeneous samples and participants are recruited through convenience sampling (Willig, 2001). The sample recruited in the current study was homogeneous. Variations in the duration and type of hair loss were controlled, and the sample was appropriate for an IPA study (Table II).

**Table I:** Summary of the key participant characteristics.

Participant pseudonyms	Age	Sex	Age of onset	Duration since diagnosis	Current academic level	Background	SES	Family history of AA
Nadeem	18	Male	15	3 years	Intermediate	Rural	Middle	Yes
Ayesha	18	Female	15	3 years	Intermediate	Rural	Middle	No
Sadia	19	Female	17	2 years	Intermediate	Urban	Upper	No
Iqbal	16	Male	14	3 years	Matric	Rural	Middle	No
Farah	17	Female	16	1 year	Matric	Urban	Middle	No
Ali	16	Male	15	1 year	Matric	Urban	Upper	Yes
Maira	19	Female	16	3 years	Bachelors	Rural	Lower	No
Heena	17	Female	15	2 years	Intermediate	Urban	Upper	Yes

Participants were asked to self-report their socio-economic status (SES) as upper, middle, or lower. Matric=10 years of education; intermediate=12 years of education; bachelors=14 years of education.

**Table II:** Master table of themes.

Superordinate themes	Themes	Subthemes/subordinate
Loss (self/social)	Loss of self	Loss of self-esteem
		Loss of self-confidence
	Social loss	Loss of love from friends
		Loss of love from family
		Left old friends
		Left going to college
		Left company of neighbours
		Being mocked by friends
Concerns (physical/future)	Physical concerns	Look less attractive
		Look older
		Look ugly
	Future concerns	Can never get married
		Can never get love
		It will get worse with time
		Will have to live alone rest of my life
		Will be ridiculed throughout my life
		Never gain back lost self-confidence.
Negative (emotions/thoughts)	Negative emotions	Anxiety
		Envy
		Jealousy
		Distress
		Shame
		Guilt
		Depression
	Negative thoughts	Hurt myself
		Hurt someone
		Worthlessness
Coping styles (adaptive/maladaptive)	Adaptive: action-oriented coping	Use of oil
		Use of homeopathic medicine
		Use of allopathic medicine
		Use of herbal medicine
		Tibb traditional medicine
		Use of dessi medicine
		Use of homemade remedies
	Practical coping	Wearing veils
		Wearing scarf
		Wearing hats
		Changed hairstyle to cover baldness
	Self-distraction	Spend most time studying
		Sports/cricket
		Started watching movies
		Started making new friends
		Started visiting friends more than before
	Religious coping	Started reading the Holly Quran
		Started reading religious books
		Started attending religious congregations
		Started offering prayers
		Started attending religious rituals
		Prayed to God to solve my problem
	Future practical coping	To earn money and get hair transplanted
		To persuade parents to get hair transplanted
	Support seeking	Support seeking from friends
		Took support from mother
	Acceptance	God's will
		Fate and one has no control
	Humourous coping	I make fun of myself in front of friends
	Maladaptive: blaming	Felt angry and blamed God
		Blamed my luck/fate
		I think I am unlucky
	Intropunitive avoidance	Stopped looking in the mirror
		Stopped wearing fashionable/new clothes
		Stopped going for shopping
		Stopped going to weddings
		Started cigarette smoking
		Stopped meeting old friends

#### Interviews

This approach to interviewing is an attempt to implement IPA's inductive epistemology to the fullest extent. The resulting interaction is defined even more by the participant and is not structured around *a priori* issues or researcher led assumptions or topics. The procedure adopted in this study involved constructing a schedule to help prepare for possible semi-structured interviews, thinking how the interview might go, and how the interviewer might respond to the participants’ responses. This approach to interviewing is an attempt to implement IPA's inductive epistemology to the fullest extent. The common approach adopted by the IPA researcher is to collect data from semi-structured interviews where the interviewer has developed a few main themes for discussion with the participants and some prompters.

An unstructured approach to interview was adopted to limit the possible danger of analysis merely reflecting the key topics identified within the interview schedule. It capitalizes upon IPA's ability to explore unanticipated and unexpected findings.

A semi-structured interview schedule was designed and questions were phrased in such a way to move from general issues to more particular ones. The semi-structured interview schedule was kept flexible so that the role of the interviewer as an active listener was maintained and when required could be abandoned to follow the concerns of the participant.

Semi-structured interviews explored participants’ recollections of psychological and social ongoing experiences of living and coping with their condition. Topic areas covering the psychological and social world of the adolescents with AA were explored during the interviews. The interview schedule questions and probes were drawn from relevant research literature and further elaborated with the help of three pilot interviews. These three interviews were in addition to the eight interviews that were carried out later and are included in the analysis. The interviews focused on the person's understanding about the consequences of AA; and the interviewer took care to check and clarify where there was ambiguity or lack of clarity. The interview was conducted to facilitate rapport and empathy, and permit flexibility of coverage of the topics under investigation.

The interview schedule included open-ended questions that broadly addressed the following areas: psychological issues, social concerns, and coping behaviours.

#### Data analysis

IPA can be characterized by a set of similar processes, moving from the particular to the shared and from the descriptive to the interpretative, and principles which are applied flexibly, according to the analytic task (Reid, Flowers, & Larkin, 2005). Typically, analysis has been described as an iterative and inductive cycle (Smith, 2004).

IPA is not a prescriptive approach; rather, it provides a set of flexible guidelines which can be adapted by individual researchers in light of their research aims (Smith & Osborn, 2003). IPA approach was used to analyse data from one-to-one interviews in order to develop “thick descriptions” that could illuminate the lived psychological and social experiences of its respondents.

The study had a small sample as recommended by IPA. The individual transcripts were analysed with idiographic intensity with regard to details. The transcripts were analysed by both authors by using IPA (Smith et al., 2009). In total, 143 pages of transcripts were generated from these eight interviews. To improve the overall validity of the results, two raters (both authors) coded the transcripts (Miles & Huberman, 2005). This interpretative process is achieved and facilitated by a series of analytic steps that enables the researcher to identify themes and integrate them into clusters.

The following steps were carried out to analyse the data (Smith et al., 2009):

*Step I: Reading and rereading*: We read and reread the verbatim and transcripts several times, imagining the voice of the participant during subsequent readings which assisted with a more complete analysis. The audio recordings were listened to while first reading the transcript. Semantic content and language use were analysed on an exploratory level. This reading and rereading facilitated an appreciation of how rapport and trust was built across an interview and helped highlight the location of richer and more detailed sections of the interview.

*Step II: Initial noting*: Once the researcher had an overall sense of the data, initial note taking was started. The transcript was set out with a wide margin down the left-hand side and lines were given numbers. The purpose was to assist with identifying examples of different themes. During note taking the semantic content and use of language on a very exploratory level was kept in consideration. Anything of interest and any striking issue within the transcript was given due attention. This step facilitated in creating familiarity with the transcript and helped identify specific ways by which the respondents talk about, understand, and perceive their lived experiences.

A descriptive core of comments, which had a clear phenomenological focus, and stayed close to the participant's explicit meaning, was highlighted during this phase. Interpretative noting helped to gain insight into the how and why of the of the participants’ concerns. The non-verbal language the participants used, thinking about the context of their concerns, and identification of abstract concepts, helped in drawing a sense of the patterns of meaning reported by the participants.

*Step III: Developing emergent themes*: Once the notes were added to the left side margin of the transcript, the process of abstraction was initiated. An attempt was made to reduce the volume of the transcript and the initial notes, though complexity, in terms of mapping the interrelationships, connections, and patterns between exploratory notes, was maintained. This involved an analytic shift to working primarily with the initial notes rather than the transcript itself. The themes were inserted in a margin on the right-hand side next to the specific section of data it related to it.

*Step IV: Searching for connection among emergent themes*: We then looked for connections between themes in order to cluster them together in a meaningful way and identify “superordinate” themes. At this step it became possible to name the “superordinate” theme by looking at what the subthemes have in common. Smith and Osborn (2003) suggest that researchers “imagine a magnet with some of the themes pulling others in and helping to make sense of them.”

*Step V: Moving to the next case*: New and unexpected themes were looked for in every case and the process was repeated for all the cases. Every transcript was considered individually, and bracketing the ideas emerging from the analysis of the first case while working on the second.

*Step VI: Looking for themes across cases*: Connections across cases, and how a theme in one case helped highlight a different case, were given consideration at this step. We tried to identify both convergence and divergence between transcripts. This led to reconfiguring and relabeling of themes, followed by drawing an integrative table of master themes across cases comprising subordinate/subthemes, themes, and superordinate themes (Table II).

## Results

The analysis resulted in four superordinate themes, 16 themes, and 66 subthemes. The four themes form a causal progression from loss (of self-esteem and identity) to concerns about physical appearance and the future, as well as negative thoughts and emotions, and the need to develop ways of coping with the loss. Two bipolar themes falling under one broad framework were combined together to depict one superordinate theme (Table II).

### Loss (self/social)

Females reported experiencing social loss, loss of self, or both, including loss of love from family and friends. Loss of self-esteem and loss of self-confidence was reported by the females whereas males reported loss of self-confidence and loss of love from friends. The key difference is that males did not report loss of self-esteem, which may reflect more identity problems for females.

Males and females both reported loss of self-confidence. They were not as confident as they were before they lost their hair. One female said:

I was so confident of myself before all this happened to me. I am no longer sure of myself. I walked confidently and sat confidently, but now I feel people can find out that I am not the same person … can't even walk straight. I have lost my confidence. [Ayesha]

One male said “I try to be confident but deep down I know I am not confident about my looks, my abilities, my relationships … nothing is the same, I am unsure about everything. I feel unworthy, ashamed and inferior. [Iqbal]

Hair loss has profound negative effects on how individuals view themselves:

I want that I should respect myself and have more positive attitude towards myself but I feel like a total failure. I don't know why I bother so much about people around me. I think it's the fear of rejection … I cannot take negative comments even from strangers. [Farah]

I feel ashamed of my looks … I think I am no longer physically appealing to the females. I have started avoiding the company of my female class fellows. [Ali]

The loss is not just about how individuals feel about themselves; it is about the loss of relationships:

After all what happened to me I feel a lot of gap between myself and my friends. Deep down I have a feeling that they no longer care for me. At times I feel reluctant visiting them. Many a times I hesitate going out with them. [Maira]

Another participant said,

I no longer go out with old friends, I am ok with new people they don't ask questions and above all they know me as I am not as I was before. I have stopped playing cricket with my neighbors. It's not that I don't want to play, it's just that I no longer feel comfortable playing. [Iqbal]

### Concerns (physical/future)

Males were concerned about looking older; females were worried about looking ugly without hair. People also worry about physical changes “I feel uncomfortable with people who knew me before this happened,” including “I know I look much older and usually my friends comment and call me dad, grandpa” [Nadeem] and “I have got used to the ridicule and now try to take it easier. I know I will have to bear the ridicule throughout my life. It's the culture … cannot stop people from making fun of the baldies” [Iqbal].

Females reported a number of future concerns and the most apparent one was the fear of not being able to fall in love or get married because of AA; which would mean a life alone. According to one participant, “I know I will have to live alone throughout my life. Who is going to marry a bald girl? No one will ever like me as a partner … no one … I am mentally prepared for it” [Heena].

### Negative emotions/thoughts

Only the females reported negative emotions such as being ashamed of their AA, feeling guilty and depressed. Females tended to discuss thoughts of hurting themselves whereas males discussed thoughts about hurting someone else. Thoughts about being worthless were common to both. One female participant said “When I see females with long thick hair deep down I get jealous. I start comparing myself with other females who have hair” [Saadia].

I know my worth is not in my hair but still there are many times I feel worthless. I think if I had a full head of hair I would be a different person. These feelings of worthless at times make me so unhappy. [Ayesha]

When my friends joke and make fun, I know they don't do it to hurt me but inside it bothers me and sometimes makes me angry. I usually don't show my anger to them. Once I couldn't control my anger and slapped my friend's cousin who ridiculed me. I can't take jokes from mere acquaintances. [Ali]

### Coping (adaptive/maladaptive)

Coping was expressed in both maladaptive and adaptive themes such as blaming, intropunitive coping, action-oriented coping, practical coping, self-distraction, support seeking, religious coping, acceptance, humour, and future practical coping. A unique, hierarchical, and overlapping pattern of coping behaviours that emerged over the period of time was seen in all the adolescents.

#### Maladaptive coping (Blaming)

Different manifestations of coping behaviours were reported by both sexes. Blaming was a coping behaviour they used just after the initial diagnosis. Females felt angry and blamed God or their fate; males blamed fate and luck, so it was not under their control. One female participant said “My first reaction was very strong. I blamed God for all this, I was angry with him. I kept questioning him why he was so cruel to me … I held him responsible for my condition” [Heena].

Another male participant said,

I think its sheer bad luck. I have never been lucky … but all is out of my control. There is a higher power out there who has the remote control. So no use getting upset for things that are destined to happen. [Nadeem]

#### Intropunitive coping

Females used intropunitive avoidance behaviours; they stopped going to social occasions, stopped meeting old friends, stopped looking in the mirror, and stopped wearing fashionable clothes. One female said she has stopped going shopping and stopped going to college “Oh I hated my looks, I stopped buying new clothes. For two years I never went shopping” [Saadia].

The only intropunitive avoidance behaviour reported by males was increased use of cigarettes “I used to smoke even before I got alopecia but after I lost my hair I became a chain smoker … I got relief from smoking” [Iqbal].

#### Adaptive coping (action-oriented coping)

Action-oriented coping included trying different types of treatments. Females tended to try hair oil, herbal, and traditional medicines, whereas homeopathic and allopathic medicines were used by both sexes. One participant said, “I tried all sorts of traditional formulas thinking they will work. I kept using them for many months then shifted to homeopathic medicine. Nothing worked … not even what dermatologists prescribed” [Maira].

#### Practical coping

All adolescents reported that after trying these treatments and failing to recover their hair, all of them stopped using treatments and tried to hide their hair loss. Females started covering their heads by wearing veils and scarfs, and the males initially tried to hide the bald patches by changing their hairstyles but later when the bald area grew in size, started wearing caps. According to one participant, “I was left with no choice but to cover my head. I have never done it before, it seemed so difficult … I looked in the mirror and just did not like my looks. I looked so odd” [Ayesha]. One male participant shared, “At the start it was easy to hide the bald patch in the center. I would just keep it long, and comb my hair from one side across to the other side” [Ali].

#### Self-distraction

Self-distraction provided some relief. Females reported that they started spending most of their time studying, whereas males said they started spending more of their time playing sports such as cricket, watching movies, making new friends, and starting to visit friends more than before.

I was always in the library studying; I suppose I did not want to face the world out there. I got very good grades. I felt comfort in studying; it kept me away from all the thoughts that could bother me. [Farah]

#### Support seeking

Sex difference in the use of support-seeking behaviours was evident; males got support from friends whereas females acquired social support from their mothers. The only support I had was from my mother, “My mother was always there for me through thick and thin. If it wasn't for her, life would have become really hard” [Saadia].

#### Religious coping

Reading the Quran and other religious books, attending religious congregations, offering prayers, attending religious rituals, praying to God and requesting to solve the problem were frequently used strategies by females. Males coped by praying to God and becoming more consistent in offering prayers five times a day. One female participant said, “The only support I got was from my connection with the God. I used to pray and at times talk with him. I prayed to him to solve my problem” [Farah]. Another male participant said, “My daily routine changed. I started offering all the five prayers. My daily routine revolved around prayers and reciting Holy Quran, all this relaxed me” [Nadeem].

#### Acceptance

Acceptance was common to both males and females but this strategy generally appeared after other types of coping behaviours had been tried out. Males were more of the opinion that their AA was God's will, whereas females felt that it was their fate and they had no control over it. One participant stated, “I am Ok with it, it's better than getting a terminal illness. It's God's will, he has a plan for all of us and we can do nothing” [Iqbal].

#### Humour coping

This was typical of males. They reported that they made fun of themselves in front of friends before their friends could joke and make fun of them. Humour was not a coping behaviour reported by females.

I don't mind jokes from friends and family. I have become more hilarious, I enjoy joking about bald people. It's a part of our culture … so I no longer mind even cracking jokes and making fun of my baldness. [Nadeem]

#### Future practical coping

Future practical coping was common in males and females. Males said that they would earn money and get their hair transplanted whereas females said that they would persuade their parents to get them hair transplanted. Proactive coping behaviours initiated at a later stage. Two of the adolescents said they had planned to get their hair transplanted. One of them said, “I want to become a businessman, earn money and get my hair transplanted from a technically advanced country” [Ali].

Another participant stated,

The only hope which keeps me going is that one day I will be able to persuade my parents to get me a hair transplant. This is the only wish I have; I have faith in God it will get fulfilled one day. [Ayesha]

## Discussion

This study shed light on the lived experiences of adolescents with AA regarding their psychological and social concerns and coping behaviours. The four themes that emerged together form a causal progression from loss (of self-esteem and identity) to concerns about physical appearance and the future, as well as negative thoughts and emotions, and the need to develop ways of coping with the loss. A unique, hierarchical, and overlapping pattern of coping behaviours that emerged over the period of time was seen in the adolescents. Initial maladaptive forms of coping behaviours were later replaced by more adaptive forms of coping: blaming God and fate, intropunitive avoidance (maladaptive), use of remedies/treatments, practical coping behaviours, self-distraction, support seeking, religious coping, and acceptance (adaptive). A series of psychological adjustments are necessary before one starts reconciling to the changed appearance (Papadopolous & Bor, 1999). The results of this study are in accordance with the three pattern framework of body image coping (avoidance, appearance fixing, and acceptance) (Cash et al., 2005).

The physical and psychological changes resulting from hair loss may lead to many psychosocial concerns and challenges. Females recounted greater feelings of loss of love from family and friends whereas males reported only loss of love from friends. Social loss, such as loss of love from friends and family; having left old friends and company of neighbours; and stopping going to college were reported by the participants.

Social avoidance and withdrawal can be a common response to disfigurement and in turn may lead to smaller networks of support from family and friends (Newell, 2000). Newell and Marks (2000) suggest that psychological and social difficulties of people with physical concerns are comparable to those with social phobia. People with visible physical disfigurements can face difficulties in social encounters, and these difficulties may lead to social withdrawal and isolation (Rumsey, 2002).

Adolescence is a critical period for the development of self-esteem. Adolescents who think their looks determine their self-worth have lower self-esteem and higher level of depression. Warm, supportive parenting is associated with high self-esteem (Erol & Orth, 2011). Females in this study reported loss of love from family and friends after the onset of AA. If family and friends do not think highly of them, they will in turn start experiencing loss of self-esteem. Altered social relationships that emerge from physical illness or change add to an eroded sense of self-esteem and self-worth (Penninx et al., 1996). This may involve a spiral of negative emotions (social anxiety); negative thoughts (fear of negative self-evaluation and negative self-perception); lowered self-esteem, confidence, and negative behaviours (social avoidance); and avoidant behaviour by others.

Females reported psychological disturbance which could be related to the greater social emphasis placed on females’ appearance (McGarvey et al., 2001). Adolescents feel uncomfortable revealing their appearance-related apprehensions to peers which further leads to feelings of isolation and social avoidance (McKillop, 2010). Pearlin and Lieberman (1979) coined the term “reflected appraisal.” They suggested that people see themselves as their significant others perceive them. These perceptions may be erroneous, but they are important to the person. Females are judged more than males on their appearance. If they deviate from cultural and social standards of beauty, people think poorly of them, and through “reflected appraisals,” based on socially constructed ideals of feminine beauty, their self-esteem may get affected.

Females reported feelings of guilt, shame, and embarrassment, which are associated with low self-confidence and self-esteem (Else-Quest, Higgins, Allison, & Morton, 2012). Self-esteem for teenage females and adolescents is associated with social ideals of beauty, femininity, and assessment of body (Abell & Richards, 1996). Although males and females start with comparable levels of self-esteem in early adolescence (between ages 11 and 13), they diverge through the teenage years and adulthood with males having a greater sense of positive self-esteem and females losing that sense (Rosenfield, 1999).

Males reported a loss of self-confidence but no loss of self-esteem. Physical appearance and perceptions of attractiveness are likely to be more important for judgments of self-worth for women than men.

In the present study adolescents reported loss of self-confidence. AA is a disfiguring disorder and issues relating to self and identity are likely to arise. Visible disfigurement can result in lowered self-confidence and negative self-image. Concern about physical looks was evident among all the eight adolescents interviewed in the present study. Concerns about looking older in males and looking ugly without hair in females were common. Men usually are expected and socialized to be less worried over their looks than women (Pope, Phillips, & Olivardia, 2000).

Females reported a number of future concerns and the most apparent one was the fear of not being able to get married because of their hair loss. Related concerns were about getting love, having to live alone for the rest of their lives, being ridiculed throughout the life, and fear of never getting back their lost self-confidence. Hair loss is a traumatic experience for both men and women, but it is “significantly more distressing for women” (Matuszek, Nelson, & Quick, 1995) and distressing reactions can occur irrespective of the type of hair loss. Cash et al. (2005) found that though men regard hair loss as undesirable and are embarrassed, they can cope and maintain integrity of their body image, as male baldness is usually accepted and relatively more common.

Males were concerned about their alopecia getting worse with the passage of time and not being able to get back their lost self-confidence. They did not convey psychological concerns such as not being able to get married or getting love. Unpredictable patterns of hair loss can result in feelings of apprehension, uncertainty, and feeling unable to control one's appearance and get back self-confidence (Cash et al., 2005). Men have fewer psychosocial concerns when faced with a physically altering condition (Pope et al., 2000).

Another superordinate theme that emerged was experiencing negative cognitions and emotions. Constant negative thoughts and language are likely to create anxiety, worry, and self-blame, which could be classified as negative forms of coping.

Thoughts about hurting one self, hurting someone else, and thoughts of being worthless were common. Alopecia, which was not surprising as AA has negative psychological repercussions (Hunt & McHale, 2005), manifested in the form of anxiety, depression, lower self-esteem, and poorer quality of life (McGarvey et al., 2001).

Coping mechanisms changed and improved with time. Initial maladaptive forms of coping behaviours emerged such as blaming God, fate, and intropunitive coping; and were latter replaced by more adaptive forms of coping such as use of remedies and treatment, wearing of veils and caps (practical coping), self-distraction, support seeking, religious coping, acceptance, humourous coping, and future planning coping.

Furthermore it takes time to adjust to the unpredictable nature of the illness, and development of more adaptive behaviours takes place at a later stage (Papadopolous & Bor, 1999). A series of psychological adjustments are necessary before one starts reconciling with the changeable appearance (Papadopolous & Bor, 1999). The results of our study are in accordance with the three pattern framework of body image coping (avoidance, appearance fixing, and acceptance) (Cash et al., 2005).

Females reported use of intropunitive avoidance; they stopped going to weddings, stopped meeting old friends, stopped looking in the mirror, and stopped wearing fashionable clothes. Males said they started smoking. The intropunitive avoidance can be explained by Thompson, Kent, and Smith (2002) model based on cognitive behavioural principles. Anxiety related to appearance is likely to result from perceived stigma caused by social norms. This anxiety increases when an individual has to face a “triggering event,” leading them to assume two ways of coping intended at reducing their anxiety (specifically, avoidance and concealment). Use of homeopathic and allopathic medicines was reported by the adolescents. Females reported use of hair oil, herbal, and traditional medicines. After trying out these treatments and failing to regain their hair, females started covering their heads by wearing veils and scarfs. Males initially tried to hide the bald patches by changing their hairstyles and later when the bald area grew in size they started wearing caps. Planning or actively coping with a diagnosis (or relapse) of AA may mean that individuals may choose to wear hair pieces and head covers such as wigs, scarves, beanies, and hats, as an effective way to deal with visible hair loss (McKillop, 2010).

Self-distraction provided relief; females reported that they started spending most of their time studying, whereas males started playing sports such as cricket. Distraction as a coping strategy has been recognized as being used by adolescents in a more sophisticated manner (Zimmer-Gembeck & Skinner, 2011). Males play sport and females turn to other distractive activities when having to face a stressful experience (McKillop, 2010).

Use of support-seeking behaviours was evident, males got support from friends and females acquired social support from their mothers. Literature proposes that females are better in receiving support from family and close friends (McKillop, 2010). Support seeking from family as a coping behaviour is effective for some people especially women with AA (Prickitt, McMichael, Gallagher, Kalabokes, & Boeck, 2004). A greater reliance on sources of social support (beyond parents) is evident during adolescence in males (Zimmer-Gembeck & Skinner, 2011).

Recitation of the Holy Quran, reading of religious books, attending religious congregations, offering prayers, attending religious rituals, praying to God, and requesting to solve the problem were frequently used religious coping strategies by females. Males coped by praying to God and getting more regular in offering prayers five times a day. God is considered as a safe haven in emotionally stressful situations (Mikulincer & Shaver, 2007).

Coping using humour was typical to males and they reported that they made fun of themselves in front of friends before their friends could joke and make fun of them. Humour may be beneficial when and if an individual has accepted their condition and is feeling very positive about their situation. Humour coping evolved at a later stage once the males had indulged themselves in religious coping and accepted their physical change. None of the females stated the use of humour. It is found out that males in face of a permanent stressor usually turn to humour, or hobbies such as sports (Plancherel & Bolognini, 1995).

The use of future practical coping was common among the adolescents with AA. The males said that they would earn money and get their hair transplanted whereas females said that they would persuade their parents to get them hair transplanted. Proactive coping behaviours were initiated at a later stage. Men and women differ in the use of coping strategies, with males assuming more problem focused strategies and females adopting a more emotion focused approach (Ptacek, Smith, & Dodge, 1994). Some of the sex differences are a depiction of cultural differences learned during the socialization, like males reporting control compared to females who said that they would ask their parents to get their hair transplantation. Females reported use of intropunitive avoidance behaviour as they faced more cultural pressures regarding their physical appearance.

The sample was voluntarily based and a predetermined criterion was used to include adolescents in the study. No attempt is made to generalize these results to the general population of people with AA. It is recognized that the participants might have psychosocial experiences and coping behaviours different from other adolescents with AA. Eight adolescents were included in the study; IPA studies have been conducted with one, three, four, nine, and 15 or more participants (Smith et al., 2009).

Although we need to further our understanding of the impact of AA on people of different ages and with different backgrounds, the current study provides some insight into the significant impact of AA on adolescents from Pakistan. Adolescent males and females have different psychosocial experiences and coping behaviours, so one focus of future studies should be to study the experiences of males and females separately.

We need to know the impact of different types of alopecia, their impact at different ages, across sexes, and in different cultures. Most importantly, what can psychologists do about it? Health care providers and student counsellors need to understand the negative psychosocial consequences of living with a visible disfigurement and provide appropriate psychological and social support, along with recommendations for cosmetic covering of the hair loss, at least partly because current medical treatments have limited effectiveness. Future research can be directed to determine whether clients with AA would be willing to access and use such psychosocial services.

The limited effectiveness of medical interventions means there is a need for research into the effectiveness of psychotherapy for the psychological and social problems associated with AA. Currently there is very little evidence relating to the reduction of AA-related psychological symptoms through such means. Clinicians should adapt their approaches to take account of the special needs of people with AA, especially adolescents.

Our findings highlight the potentially universal nature of psychosocial impact of AA. Evidence indicates that the significance of hair loss is similar in both the West and the East. The experiences reported by males and females are much in accord with the findings from Western cultures (Ptacek et al., 1994; Tucker, 2009). The coping behaviours reported were also similar to those reported earlier in UK samples, though with more emphasis on the use of religious coping behaviours. One difference was the use of veils and head scarves after visible hair loss, instead of use of wigs, in females; and the use of caps instead of head shaving in males was a common way of fixing one's appearance in this Pakistani sample. Traditional medicine and the use of a wide range of homemade remedies to counter hair loss were peculiar to the sample drawn from Pakistan. Most of the homemade remedies and traditional medicines reported by these adolescents with AA have not been reported earlier by the researchers conducted in the West.

## Conclusion

IPA is based upon the principles of hermeneutics, phenomenology, and idiography. This study is hermeneutic in understanding the recounted psychosocial experiences of adolescents with AA. It is phenomenological in recognizing and “giving voice” (Larkin, Watts, & Clifton, 2006) to the psychological and social concerns of adolescents with AA. Finally, it is idiographic in that assumptions were drawn about the experiences of the eight participants rather than that for the overall community of adolescents with AA.
